# Imperfect Duplicate Insertions Type of Mutations in Plasmepsin V Modulates Binding Properties of PEXEL Motifs of Export Proteins in Indian *Plasmodium vivax*

**DOI:** 10.1371/journal.pone.0060077

**Published:** 2013-03-29

**Authors:** Manmeet Rawat, Sonam Vijay, Yash Gupta, Pramod Kumar Tiwari, Arun Sharma

**Affiliations:** 1 Protein Biochemistry and Structural Biology Division, National Institute of Malaria Research (ICMR), Dwarka, New Delhi, India; 2 Department of Microbiology and Molecular Biology, National JALMA Institute for Leprosy and Other Mycobacterial Diseases (ICMR), Agra, Uttar Pradesh, India; 3 Centre for Genomics, Molecular and Human Genetics, Jiwaji University, Gwalior, Madhya Pradesh, India; 4 Department of Internal Medicine, University of New Mexico School of Medicine, Albuquerque, New Mexico, United States of America; UCSF/VA Medical Center, United States of America

## Abstract

**Introduction:**

Plasmepsin V (PM-V) have functionally conserved orthologues across the *Plasmodium genus* who's binding and antigenic processing at the PEXEL motifs for export about 200–300 essential proteins is important for the virulence and viability of the causative *Plasmodium* species. This study was undertaken to determine *P. vivax* plasmepsin V Ind (*Pv*PM-V-Ind) PEXEL motif export pathway for pathogenicity-related proteins/antigens export thereby altering *plasmodium* exportome during erythrocytic stages.

**Method:**

We identify and characterize *Plasmodium vivax* plasmepsin-V-Ind (mutant) gene by cloning, sequence analysis, in *silico* bioinformatic protocols and structural modeling predictions based on docking studies on binding capacity with PEXEL motifs processing in terms of binding and accessibility of export proteins.

**Results:**

Cloning and sequence analysis for genetic diversity demonstrates *Pv*PM-V-Ind (mutant) gene is highly conserved among all isolates from different geographical regions of India. Imperfect duplicate insertion types of mutations (SVSE from 246–249 AA and SLSE from 266–269 AA) were identified among all Indian isolates in comparison to *P.vivax* Sal-1 (*Pv*PM-V-Sal 1) isolate. *In silico* bioinformatics interaction studies of PEXEL peptide and active enzyme reveal that *Pv*PM-V-Ind (mutant) is only active in endoplasmic reticulum lumen and membrane embedding is essential for activation of plasmepsin V. Structural modeling predictions based on docking studies with PEXEL motif show significant variation in substrate protein binding of these imperfect mutations with data mined PEXEL sequences. The predicted variation in the docking score and interacting amino acids of *Pv*PM-V-Ind (mutant) proteins with PEXEL and lopinavir suggests a modulation in the activity of *Pv*PM-V in terms of binding and accessibility at these sites.

**Conclusion/Significance:**

Our functional modeled validation of *Pv*PM-V-Ind (mutant) imperfect duplicate insertions with data mined PEXEL sequences leading to altered binding and substrate accessibility of the enzyme makes it a plausible target to investigate export mechanisms for *in silico* virtual screening and novel pharmacophore designing.

## Introduction

Malaria, a global parasitic disease, caused by *Plasmodium* species, affects approximately 500 million people throughout the tropical and subtropical countries and causes considerable morbidity and mortality with estimated 800,000 deaths worldwide each year [1]. *Plasmodium falciparum* and *Plasmodium vivax* are considered as the two important human malaria parasites. *Plasmodium falciparum*, a virulent form of malaria, is responsible for 1 to 2 million deaths annually, mostly in children under the age of 5 years. *Plasmodium vivax* is responsible for 50–60% of all malaria cases in Western pacific and South East Asian countries of which India is a major contributor to this burden [2], [3]. Although, in comparison of *P. falciparum* the deaths due to *P. vivax* are rare, however socioeconomic impact of *P. vivax* malaria is enormous [4] and several recent reports recognized *P. vivax* induced malaria as a severe and fatal malaria [5]–[9]. Furthermore, in light of the emergence of chloroquine and multidrug resistance in *P.vivax* malaria [5], [10] and emergence of *P. vivax* strains with lower sensitivity to recent antimalarial therapy [11] there is an urgent need to develop a control strategy to identify new targets for human malaria parasite *Plasmodium vivax*.

The manifestation of malaria is heavily linked to the growth and development of the virulent form of *Plasmodium* inside the infected erythrocytes. In order to overcome the host responses, *Plasmodium* remodels red blood cell architecture and machinery, allowing the export of hundreds of effector proteins beyond the parasitophorous vacuole membrane (PVM) [8]–[14]. Among the variety of *Plasmodium* effector enzymes, the family of aspartic proteases (plasmepsins) plays a key role in a wide variety of cellular processes including the export of plasmodium proteins which are essential for malaria parasite growth/survival and have been considered as promising targets for the development of novel chemotherapeutics [15]–[19].

The primary analysis of *Plasmodium falciparum* genome has led to the identification of at least 10 members of aspartic proteases (plasmepsins) family of proteins [18]. In contrast to *P. falciparum*, *P. vivax* genome sequence database analyses have shown that *P. vivax* has 7 orthologues of plasmepsins, *Pf*PM-IV–*Pf*PM-X [20]–[21]. Although similar to the *P. falciparum*, plasmepsins of *P. vivax* have also been considered as most promising anti-malarial drug targets, however because of the lack of *in vitro* culture system, the relative role of plasmepsins has not been yet fully examined in *P. vivax*. In this context, we have recently examined the structural properties and conservation of *Pv*PM-IV in *P. vivax* from Indian isolates [22].

Unlike other plasmepsins, plasmepsin V, IX and X are not located in the food vacuole and plasmepsin-V is a unique and highly specialized aspartic protease with specific localization and function [14]. Fractional and solubilization experiments have demonstrated that plasmepsin-V is an integral membrane protein and it is distinct from those previously characterized plasmepsins. Plasmepsin-V is believed to be involved in the processing of the PEXEL motif (*Plasmodium* Export Element) and is essential for protein/antigen export [23]. PEXEL, a conserved and short N-terminal amino acid motif, when cleaved and acetylated in the endoplasmic reticulum translocates proteins into the host cells [13], [24], [ and 25]. Recent studies suggest that *Pf*PM-V is a PEXEL protease, which could be a unique antimalarial drug target against *P. falciparum* infection. However, in case of *P. vivax*, such studies on the *Pv*PM-V are limited and need attention to examine the genetic, structural and functional properties.

In this study, we examined genetic polymorphism, molecular nature and structural properties of *P. vivax Pv*PM-V gene isolated from different geographical regions of India in order to determine if this export pathway are conserved in *Plasmodium vivax*. We performed an extensive *in silico* analysis to compare substrate binding with data mined PEXEL sequences from *P. vivax* exported proteins in order to develop an experimental system for studying functional modeled validation of these export processes to understand underlying effect of mutations on the activation of enzyme in ER without N- terminal processing as reported previously [14], [26]. Our molecular and *in silico* studies add support for conservation of export pathway in *P. vivax* and predict a new putative plausible mechanism of immune evasion by *P. vivax*. Our results show that a variation in antigenic processing might be a key for emergence of more virulent type strains of *P. vivax* as differential antigen profile is known to be involved in immune evasion. *Pv*PM-V based functional prediction data provides new insights into the design of new chemotherapeutic agents and diagnostic markers against malaria *vivax* infection.

## Materials and Methods

### Study Design

The study was carried out on *P.vivax* samples from different geographical regions of India to evaluate a plausible role of *Pv*PM-V-Ind gene in genetic, structural and functional terms. This study was performed in three sequential steps namely molecular (genetic diversity and phylogenetic analysis), *in silico* structural analysis, PEXEL motif selection and docking studies with known inhibitors.

The study was conducted under the protocol reviewed and approved by the Institutional Scientific Advisory Committee (SAC) and Institutional Human Ethical Committee. Written informed consent was obtained from all the volunteers prior to the collection of *P. vivax* positive blood samples and human subject's guidelines were followed. This manuscript is approved by Institutional publication committee having approval number 019/2012.

### Study area and patient selection

The present study was conducted in seven different geographical regions of India having different topographical habitats viz. Bangalore, Chennai, Delhi, Goa, Nadiad, Rourkela and Sonapur as depicted in our earlier report [22]. The Centers selected for the study also had different international exposure i.e. Delhi, Chennai & Bangalore being urban commercial centers, Goa an international tourist destination and Nadiad, Rourkela & Sonapur sub-urban cities with low migratory population flux. *P. vivax*-infected patients, who were willing to participate and fitted the enrolment criteria [22] as per our earlier report, were included in this study as per WHO protocol [27].

### *P. vivax* sample collection

*P. vivax* +ve blood samples were collected from patients (either sex) who were visiting NIMR Malaria Clinics in seven different geographical regions of India as described in our earlier report [22]. Briefly, the blood samples were screened microscopically (thick and thin smears) for the presence of *P. vivax* +ve malaria. 2–3 drops of finger prick blood from the patients having a minimum parasitaemia (0.05 to 0.5%) were collected on 3-mm filter paper (Whatman International Ltd., Maidstone, UK) as per our earlier report [22].

### DNA isolation and PCR amplification

*P. vivax* genomic DNA was extracted from the blood samples collected on filter paper using the QIAamp DNA Blood Mini Kit (Qiagen, Hilden, Germany). For PCR amplification, a set of *Pv*PM-V gene specific primers of *Pv*PM-V (5′-ATGGTCGGAGCGAGCTTGGGGCCCCCCGGT-3′ and (5′-CTACGCATCCGCGGGCGCCTTGCCCTCGGAGG-3′), were used [28] to amplify a complete gene sequence. Furthermore, another pair of specific primers targeting specific smaller segment of gene were also designed i.e. *Pv*PM-V-5.2 (5′-GGGCGTATTGGGGATGAGTCTTTC-3′ & 5′-CGTTCGTCATCTTCAATCGCTTAT-3′). The PCR products were resolved on a 1% agarose gel.

### Cloning of *Pv*PM-V gene and sequencing

*Pv*PM-V-Ind PCR amplified products were gel-purified by using QIAquick Gel Extraction Kit (Qiagen, Hilden, Germany), ligated into the P derived cloning vector (Qiagen, Hilden, Germany) and transformed into competent *E. coli* DH5α cells as per the manufacturer's recommended protocol. Positive recombinant clones were sequenced in both directions on an ABI 3730 Genetic Analyzer (PE Applied Biosystems). For sequence validation, two independent sequencing reactions of each clone were performed.

### Sequence homology and Phylogenetic analysis

All nucleotide sequences of *P. vivax Pv*PM-V-Ind isolates encoding each gene were submitted to NCBI GenBank (accession numbers **GU569930 to GU569935**) for public domain use. The verified sequences were translated to amino acid and aligned with *Pv*PM-V of different regions of India to mark functional domain region. The translated protein sequences of *Pv*PM-V-Ind were searched using the InterProScan software to identify signatures and their topology from the InterPro member databases; Pfam, PROSITE, PRINTS, ProDom, SMART, TIGRFAMs, PIRSF, SUPERFAMILY, Gene3D, and PANTHER [29]–[30].

Orthologues of plasmepsin-V were searched on KEGG-SSDB server (http://ssdb.genome.jp) in various genomes and EuPathDB Bioinformatics Resource Center (eupathdb.org). All *Plasmodium* plasmepsin-V sequences were aligned and phylogenetic tree was created to visualize gene evolution in relation to various genomes. Various orthologous of plasmepsin-V were aligned using CLUSTALW and bootstrap phylogenetic tree was generated using Unipro UGENE: Integrated Bioinformatics Tools (ugene.unipro.ru). To analyze the evolutionary direction Indian isolate sequences and Sal I reference sequences were submitted to SPRING server (http://algorithm.cs.nthu.edu.tw/tools/SPRING/) [31].

### *In silico* Molecular modeling

All sequences were modeled for 3-dimensional structure at I-TASSER server (http://zhang.bioinformatics.ku.edu/I-TASSER/) which is based on multiple-threading alignments by LOMETS and iterative TASSER simulations [32]–[33]. Structures were further analyzed using VMD software (University of Illinois) [34]–[35] and were selected on the basis of RMSD values and less than 1% deviation from Ramachandran Plot. The modeled structures were superimposed using VMD software and their active sites and over all changes were visualized. The structural models have been submitted to **Protein Model Data Base (PMDB)** for public domain use. The structural models can be accessed at PMDB through the given id i.e. *Pv*PM-V-Ind (mutant) - **PM0078211** & **PM0077433**, *Pv*PM-V- Sal-1(wild) - **PM0078212** & **PM0077434**.

### PEXEL motif selection by data mining

Various *P. falciparum* genes whose proteins are known to be exported during erythrocytic stages were used to homology search against *Pv*PM-V-Ind in *P vivax* genome at server http://plasmodb.org/plasmo/. All genes were then subjected to complex pattern search employing 3of5 server (http://www.dkfz.de/mga2/3of5/3of5.html) [36] using two separate pattern searches with formulae {[KR][GAVLIMFWPSTCYNQ][LI][GAVLIMFWPSTCYNQ][DEQ]} for true PEXEL and {[KR].[LI].[DEQ]} for loose PEXEL motif where capital letters denote individual amino acids, multiple amino acids in brackets[XZ] represent ambiguity in pattern and full stop (.) means any amino acid. Five sequences with a true PEXEL pattern and five more with loose PEXEL pattern were selected and used.

### Docking of PEXEL and inhibitor Lopinavir with *Pv*PM-V active (tail deleted) 3D models

Thirty extra amino acids, 15 upstream and 15 downstream PEXEL sequence were included in the model. PEXEL peptide 3D structures were obtained from phyre2 server (http://www.sbg.bio.ic.ac.uk/phyre2) [37]. Modeled PEXEL peptides were molecularly docked with *Pv*PM-V tail delete models. For *in-silico* interaction studies structures were submitted to the Patch Dock server (http://bioinfo3d.cs.tau.ac.il/PatchDock) [38]. Patch Dock results were refined by submitting best 100 dockings to FibreDock server (http://bioinfo3d.cs.tau.ac.il/FibreDock/) [39]. Interacting amino acid side chains were visualized with VMD software. Similarly protein models were docked with lopinavir drug PDB structure (drugbank.ca) employing patchdock followed by firedock analysis (http://bioinfo3d.cs.tau.ac.il/FireDock/) [40].

## Results and Discussion

*Plasmodium* specific proteases, mainly plasmepsin family proteins, involved in the hemoglobin degradation, have been proposed as key antimalarial drug targets. In *P. falciparum* plasmepsin-IV and plasmepsin-V has been extensively studied to examine the functional properties. However, unlike *P. falciparum* studies on plasmepsins from *P. vivax* are poorly investigated, principally due to lack of *in vitro* culture system. Therefore, in order to understand the substantial level of molecular nature and functional properties of *Pv*PM-V-Ind, in the present study we aimed to examine the genetic polymorphism among field isolates from different geographical regions of India and structural analysis of the plasmepsin-V gene of *Plasmodium vivax* (*Pv*PM-V-Ind), through combination of genomics and *in silico* bioinformatic approaches.

### *Pv*PM-V-Ind is a highly conserved single copy aspartic protease gene having imperfect duplication insertions

**Figure 1:**
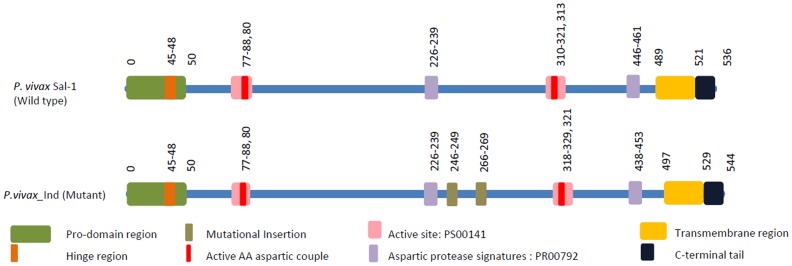
Schematic representation of various protein signatures, domains and other features of both *P. vivax* Sal-1 (wild type) and *P. vivax*_Ind (mutant) plasmepsin-V. *P. vivax*_Ind showing first imperfect duplication insertion type of mutation from 246 AA to 249 AA positions and second duplication insertion from 262 AA to 264 AA position.

**Figure 2:**
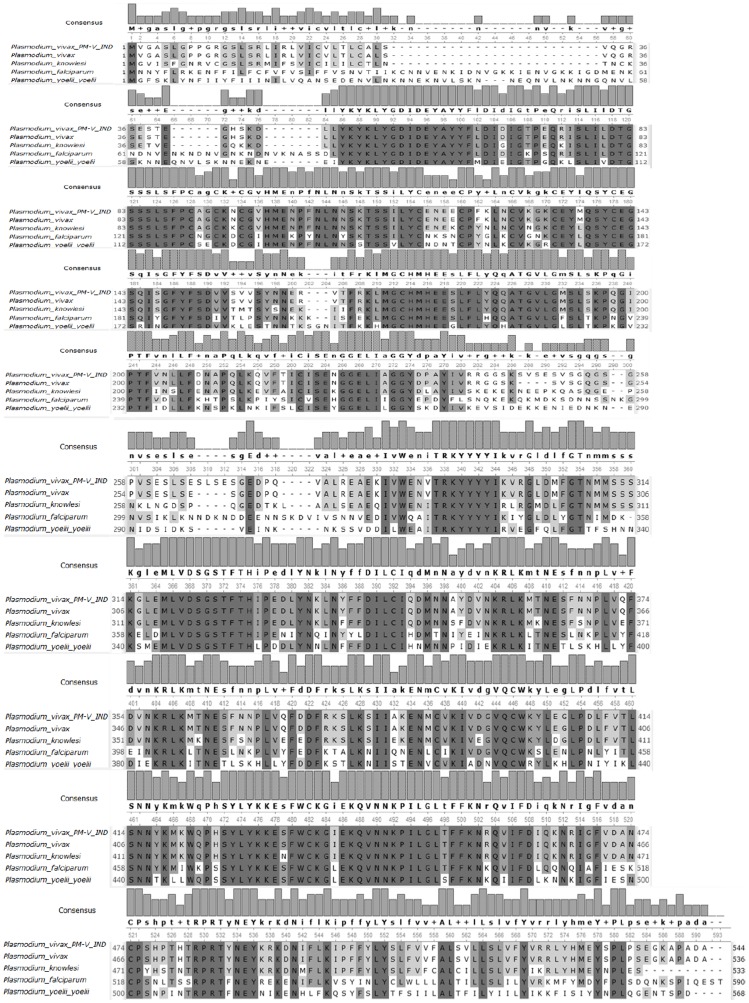
*Clustal W* multiple sequence alignment of plasmepsin-V orthologues within plasmodium genera. *Plasmodium vivax Pv*PM-V-Ind is the mutant sequence from the Indian isolates.

In order to examine the molecular nature and genetic diversity of the *Pv*PM-V, the PCR amplified products from different geographical regions of India were sequenced and analyzed through comparative bioinformatics analysis. This gene carries an open reading frame of 1635 bp, which is predicted to encode proteins of 544 amino acid residues, having a pro-domain region from 1–50 amino acids, a hing region in pro-domain from 45–48 AA, active side pocket from 77 to 88 and 318 to 329 AA with aspartic residue at 80 and 321 AA, aspartic protease signature at 226 to 239 and 438 to 453 AA, Tran-membrane domain from 497 to 529 AA and C-terminal tail from 529 to 544 amino acids (Fig. 1 and Fig. 2). We were unable to notice any polymorphism among any sequenced Indian isolate for the *Pv*PM-V-Ind gene which showed a 100% amino acid sequence identity, indicating high degree of conservation among them. However, when we compared *Pv*PM-V-Ind sequence with available *P. vivax* Sal-1 isolate which was used as control, interestingly we observed two unique mutations comprising insertions of three neutral and one acidic amino acids viz. SVSE from 246 to 249 positions and duplications of four amino acids i.e. SLSE from 266 to 269 positions, in all the Indian isolates tested (Fig. 1 and Fig. 2). The omnipresence of these mutations in *Pv*PM-V-Ind gene in isolates from different geographical regions of India could imply that either *Plasmodium vivax* infections are all evolved from single ancestor isolated from rest of the world or *Plasmodium vivax* with this polymorphism is more virulent and dominating emerging severe infections.

In order to measure the genetic relatedness and evolutionary events, we performed multiple sequence alignment analysis and compared *Pv*PM-V-Ind sequence with available plasmepsin-V sequences for other *Plasmodium* species in the database. *Pv*PM-V-Ind gene shows 98% homology with *P. vivax* Sal-1, 80% homology with *P. knowlesi*-strainH, 60% homology with *P. falciparum*, 58% homology with *P. yoelli*, [Fig. 2]. Plasmepsin-V multi sequence alignment analysis revealed that prodomain region of plasmepsin-V is most hyper variable among *Plasmodium* species 25–45 AA of *Pv*PM-V suggesting an absence of a conserved cleavage site while 45–65 amino acids were found to be highly conserved among all species of *Plasmodium*. Conserved and distinguished features of plasmepsin-V sequence have been summarized in the Figure 1.

**Figure 3:**
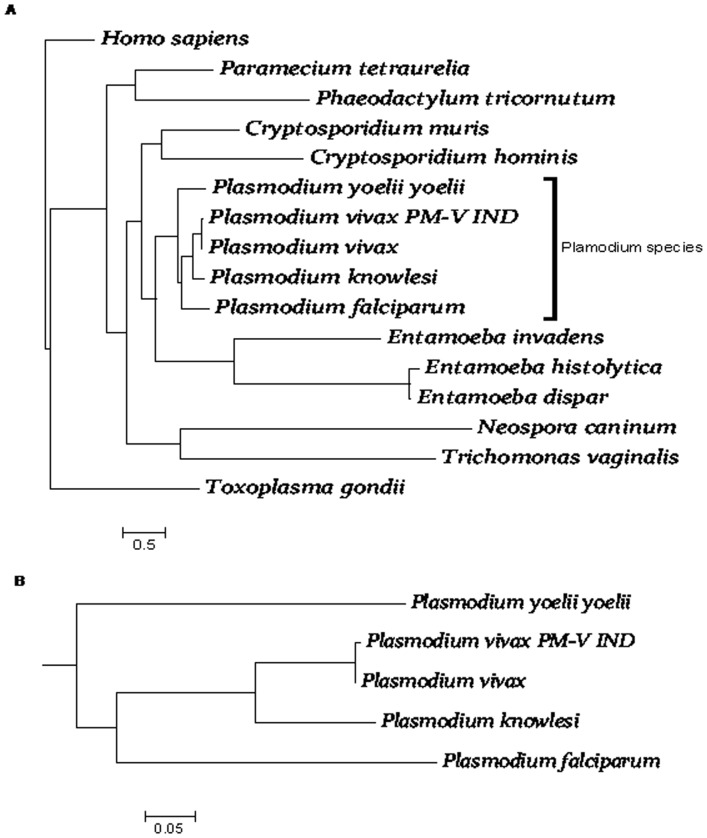
Phylogenetic analysis (bootstrap) *of* plasmepsin -V using neighbor joining method. (**A**) Different orthologue sequences of plasmepsin-V from different pathogenic protozoan. *Plasmodium vivax*_Ind (*Pv*PM-V-Ind) is the mutant sequence from the Indian isolates. (**B**) Cropped and zoomed in phylogenetic tree from Figure 3A showing Indian isolates to be a more evolved gene.

Phylogenetic analysis suggested specialized role of this gene in *Plasmodium* genome as other apicomplexan like *Toxoplasma gondi* did not share much homology with *Plasmodium* plasmepsin-V. Though the orthologues had phylogenetic relationship similar to genomic phylogeny but maximum similarity of plasmepsin-V to known orthologue was outside *Plasmodium* genera found less than 30% [Fig. 3A]. Other pathogenic protozoa did not show conservancy of plasmepsin-V orthologue in their respective genomes [Fig. 3A] however, this gene had distinct phylogeny in *Plasmodium* genera [Fig. 3B], not overlapping with other plasmepsins. Furthermore, the SPRING analysis showed that the polymorphism encountered in almost all Indian isolates is more evolved form of plasmepsin-V reported by previous workers. Sequence analysis suggests importance of this gene in *Plasmodium* genera as it is well conserved in the plasmodium genera but no conservancy with other closely related organisms.

### Functional analysis by structural modeling and protein activation prediction of *Pv*PM-V-Ind

Out of these multiple events, trafficking across the PVM essentially requires additional sequence elements named *Plasmodium* export elements (PEXEL) [13]. Recently it has been shown that aspartyl protease plasmepsin-V activities are responsible for PEXEL processing in *P. falciparum* [23]. However, unlike *P. falciparum*, the functional studies on *P. vivax* plasmepsins have been very limited, primarily due to the lack *in vitro* culture. Therefore, to predict the possible functional properties and activation pathway of *P. vivax* plasmepsin-V, in the present study we took an opportunity of the available structural and functional database of *P. falciparum* plasmepsins and compared them with the predicted molecular model of *Pv*PM-V-Ind.

In order to examine the structural and functional relationship, we first compared the modeled structure of *Pv*PM-V-Ind isolate having C-score: −2.42, TM: 0.43+0.14, and RMSD: 13.4+4.1 Å with the *Pv*PM-V Sal-1 (i.e. wild) having C-score: −2.46, TM: 0.43+0.14, and RMSD: 13.5+4 Å. This analysis showed very compact structural similarities to other aspartic proteases and the N-terminal prodomain region of *Pv*PM-V seems to block active site, enabling higher substrate specificity of *Pv*PM-V. This was consistent with both previous reports of inactivity of native protein [23] and non self cleavage activity of plasmepsin-V in *P. falciparum* [26]. *in vitro* studies on *Pf*PM-V showed activity in slightly smaller protein fragments purified by affinity chromatography with no clear mechanism of activation [23].

**Figure 4:**
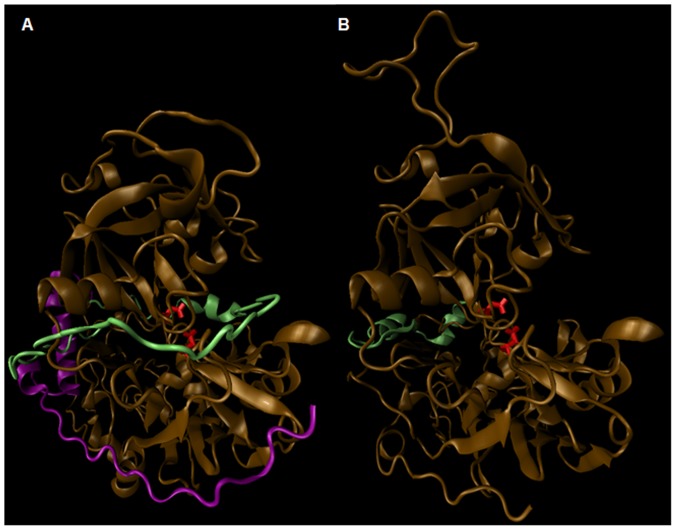
Structural cartoon representations of model of plasmepsin-V of *P. vivax*. (**A**) Complete model with C-terminal trans-membrane domain (purple), the n-terminal pro-domain (lime) inhibiting the complete active site cleft with aspartic acid residue (red) and side chains. (**B**) Displaying structural changes after the cleavage of c-terminal trans-membrane domain (purple). The N-terminal prodomain peptide (lime) frees complete active site cleft after folding at hinge and competitively interacts with same amino acid residue side chains of the model as that of C-terminal in the native structure.

Therefore, in order to predict a model with more *in vivo* similarity where it could be embedded in the membrane; the trans-membrane domain in C-terminal tail was cleaved off from the main model in functional protein. The resulting models (*Pv*PM-V Sal-1 (i.e. wild type); C-score: −2.66, TM: 0.46+0.15, RMSD: 12.3+4.3 Å and *Pv*PM-V-Ind (i.e. mutant); C-score: −2.43, TM: 0.45+0.15, RMSD: 12.6+4.3 Å) showed an overall effect of trans-membrane domain removal, enabling more stable than complete sequence models. In comparison to complete sequence model the most significant and consistent change observed in both tail deleted *Pv*PM-V Sal-1(wild type) and tail deleted *Pv*PM-V-Ind (mutant) was folding of N-terminal pro-domain region at a hing region (45–48 amino acids) and thus freeing active site pocket for substrate binding [Fig. 4A and 4B]. The N-terminal prodomain sequence after folding at hing competitively interact with same amino acid residue side chains of the model as that of C-terminal in the native structure (Fig. 4b). The pocket formed by amino acids : ‘67 ASP,72 GLU,74 ARG,223 GLU,224 ASN,227 GLU,236 ALA,243 GLY,245 LYS,248 SER,250 GLN,394 TYR’ on side chains of model acts as a common binding site at which C-terminal tail amino acids : ‘505 LEU,507 VAL,493 PHE,479 ASN,476 ARG’ binds in complete sequence model and as the C-terminal tail domain is cleaved same pocket binds with N-terminal residues : ‘30 LEU,33 GLN,35 ARG,37 GLU,40 GLU,51 LYS’ of the tail deleted model.

The prodomain folded at 45–48 amino acids, a hinge region predicted by ‘HingeProt’ server (http://www.prc.boun.edu.tr/appserv/prc/hingeprot/) [41], [44] was highly conserved among plasmepsin-V in all *Plasmodia* compared in the present study. Russo et.al [14] pulled down hsp70 of ER with recombinant protein which could be involved in re-shuffling of prodomain to free active site in membrane embedded protein [14]. Earlier studies [26] in *P. falciparum* plasmepsin-V showed the presence of plasmepsin-V in ER as well as in cytosol, while the activity was shown specifically in the ER only [24]. Taken together all this information, it can be postulated that embedded C-terminal protein part in the membrane, brings about some structural changes mediated by ER chaperons rendering it active. Russo et al [14] has also showed the activity with uncleaved GFP tagged protein while in contrast Boddey et al [23] failed to show activity in native protein suggesting GFP tag interferes in pro-domain structure and somehow frees active site, indicating that prodomain may be involved to inhibits enzyme function and as per other reports, protein may becomes activated without self prodomain cleavage once embedded in the ER membrane.

### Mutations in *Pv*PM-V-Ind shows significant effect on PEXEL motif substrate specificity and known inhibitor binding

**Figure 5:**
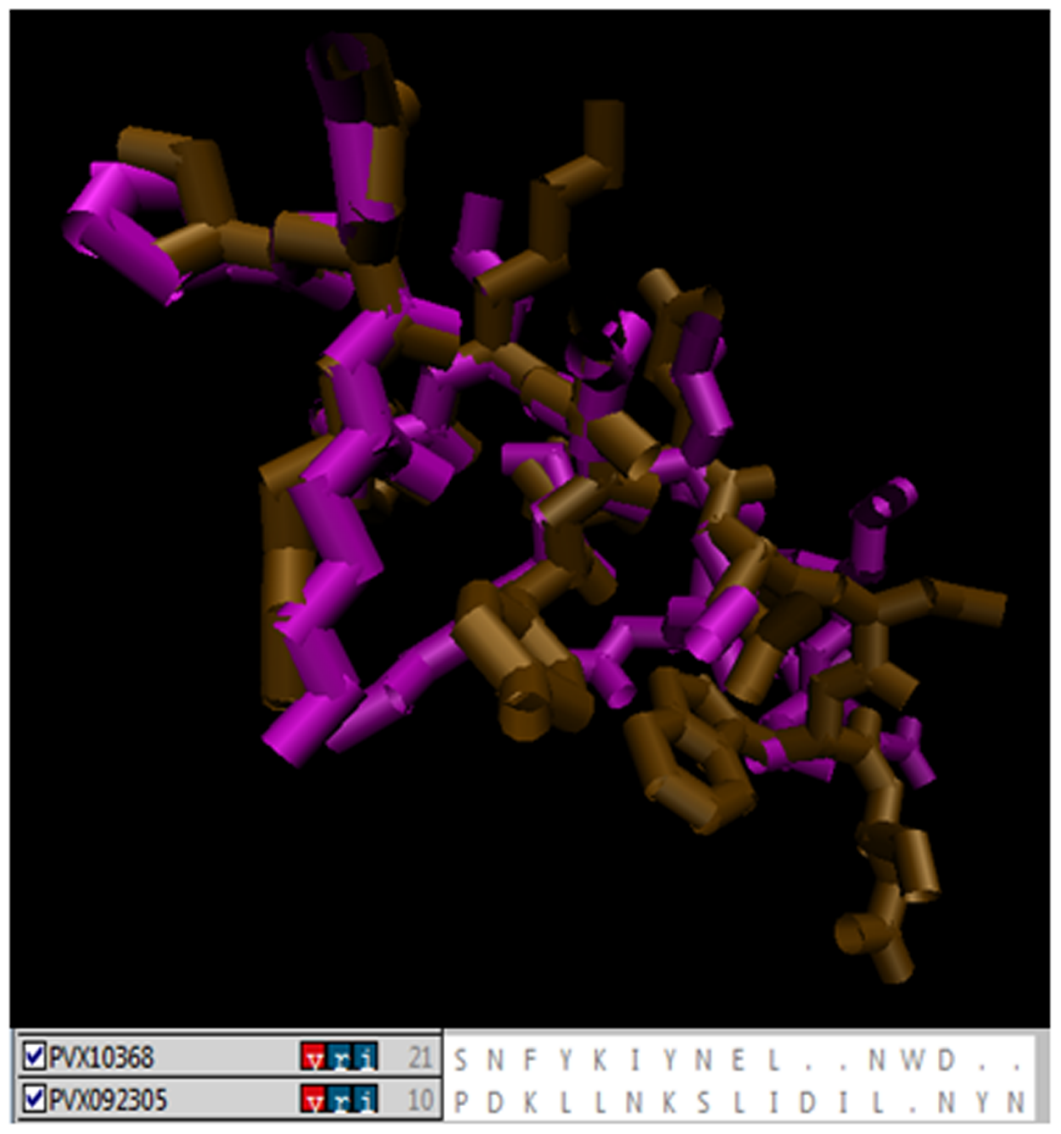
Structural alignment of PEXEL domains. Showing high structural consensus in the side chain topology in even dissimilar sequences. Brown PVX10368 (tsekdfsvdkikeeyKFIEDsnfykiynelnwdcn) PEXEL sequence and Purple PVX092305 (ksedlpskvpdkllnKSLIDilnynfnvndvmgif) PEXEL sequence.

**Figure 6:**
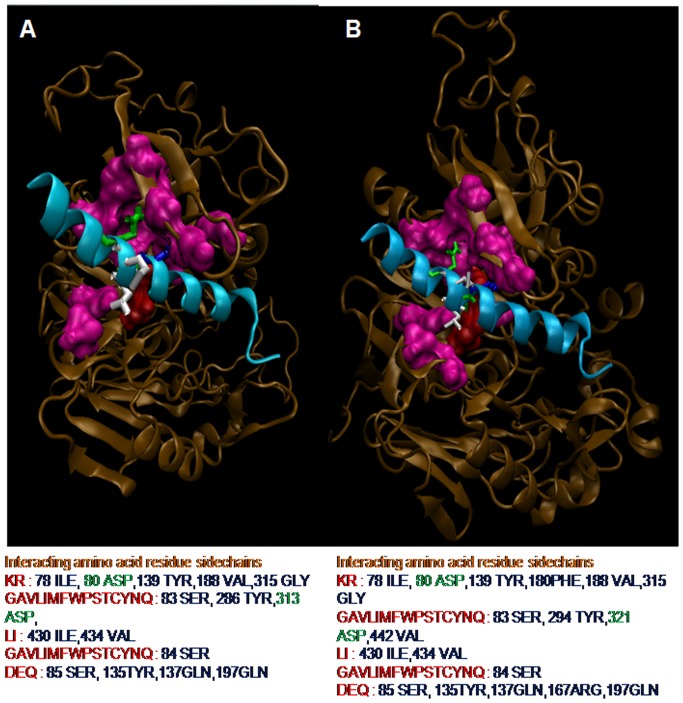
Structural representations of model *Pv*PM-V. (**A**) *Pv*PM-V Sal-1 (Wild Type) (**B**) *Pv*PM-V-Ind (mutant). Displaying docked PEXEL motif (sky blue helix) with the active site showing different pockets of interaction with different PEXEL amino acid side chains. Deepest pockets for are for first (green) and last (blue) AA. Low number of interacting AA (white) suggests more ambiguity allowed at the PEXEL member. Active aspartyl residues (red) clearly interact with the backbone of docked peptide at the point of cleavage.

**Figure 7:**
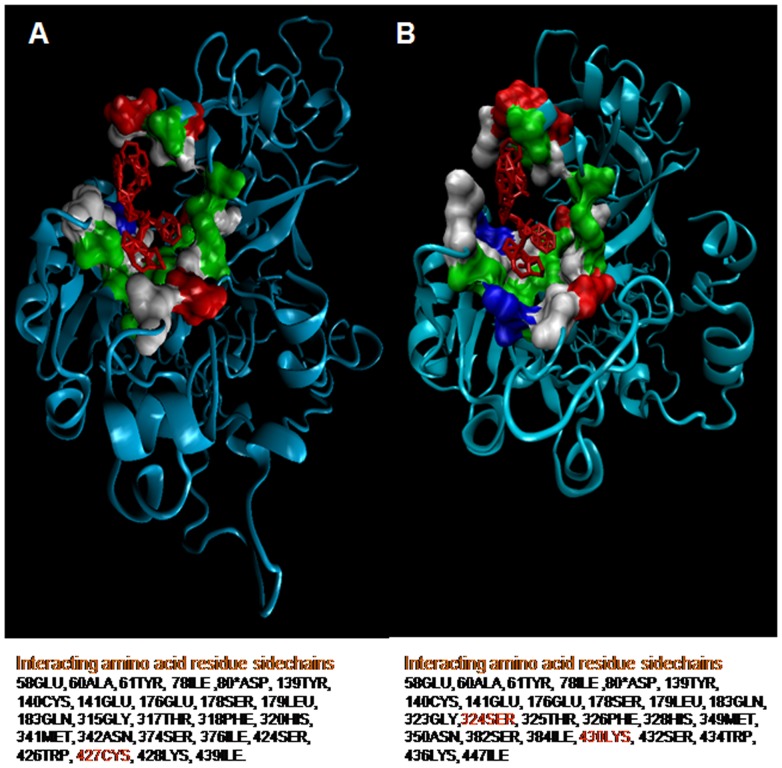
Structural representations with Lopinavir interactions. (**A**) Displaying active site of the *Pv*PM-V Sal-1 (wild type) and its interaction with only known inhibitor lopinavir (red) (**B**) Displaying active site of the *Pv*PM-V-Ind (mutant) and its interaction with known inhibitor lopinavir (red). The docked lopinavir (red) showing interaction with hydrophobic amino acid residue (white) of the active site, while hydrophilic residues: acidic (red), Basic (blue) and neutral (green) comprise of the docking site. There are clear overall changes in structure as well as in interacting amino acids with the ligand.

Next, we attempted to predict the possible involvement of two unique insertions/mutations on the substrate binding activity of *Pv*PM-V-Ind. The plasmepsin-V structure shows that the active site is quite large and canal like for binding peptide substrates. Therefore, we predict that a small change in the sequence may affect active site architecture, thereby modifying binding and processivity of resulting enzyme. As PEXEL peptides are known substrates of plasmepsin-V therefore *in silico* molecular interaction studies of PEXEL peptide and active enzyme may reveal differences in the active site/substrate binding domains. Although, PEXEL peptides have low sequence similarity among themselves, however they always tends to form a right handed helix which could be structurally superimposed displaying high similarity [Fig. 5]. Modeled PEXEL peptides docked in central canal like active domain showed clear interactions between amino acids of PEXEL peptide and amino acids of the active domain of *Pv*PM-V explaining sequence specificity of plasmepsin-V [Fig. 6A and 6B]. The target site of cleavage i.e. between third and fourth AA of PEXEL motif clearly exposed to active aspartyl side chains [Fig. 6A and 6B]. Docking analysis showed unique changes/variation in the interacting amino acids (Table 1). Further, in order to compare the *Pv*PM-V-Ind (mutant) and *Pv*PM-V Sal-1 (wild) active sites, the comparative docking scores of all the five tight PEXEL [Table 2] and lose PEXEL were tabulated [Table 3]. The docking scores of *Pv*PM-V-Ind (mutant) and *Pv*PM-V Sal-1 (wild type) clearly shows a lot of variation in Global energy as well as in ACE scores in case of both tight as well as loose PEXELs. In order to further analyze the effect of insertion/mutation on the active site domain of *Pv*PM-V-Ind, only known inhibitor of plasmepsin-V i.e lopinavir [14] was molecularly docked with plasmepsin-V tail delete models [Fig. 7]. Docking analysis again showed unique changes/variation in the interacting amino acids (Table 1) and docking scores for *Pv*PM-V Sal-1 (wild type) and *Pv*PM-V-Ind (mutant) structures (Fig. 7a & 7b). Therefore, the predicted variation in the docking score and interacting amino acids of *Pv*PM-V Sal-1 (wild) and *Pv*PM-V-Ind (mutant) proteins with both PEXEL and lopinavir suggests a modification in the activity of *Pv*PM-V-Ind might have resulted from this mutation.

**Table 1:** The predicted variations in the interacting amino acid residues side chains of *Pv*PM-V Sal-1 (wild) and *Pv*PM-V-Ind (mutant) with PEXEL and only known inhibitor Lopinavir.

Legend	Interacting amino acid residues side chains for *Pv*PM-V Sal-1 (Wild)amino a	Interacting amino acid residues side chains for *Pv*PM-V- Ind (Mutant)
**PEXEL**	**KR**: 78 ILE, 80* ASP, 139 TYR, 188 VAL, 315 GLY.**GAVLIMFWPSTCYNQ**: 83 SER, 286 TYR, 313* ASP.**LI**: 430 ILE, 434 VAL.**GAVLIMFWPSTCYNQ**: 84 SER.**DEQ**: 85 SER, 135TYR, 137GLN, 197GLN.	**KR**: 78 ILE, 80* ASP, 139 TYR, 180 PHE, 188 VAL, 315 GLY.**GAVLIMFWPSTCYNQ**: 83 SER, 294 TYR, 321* ASP, 442 VAL.**LI**: 430 ILE, 434 VAL.**GAVLIMFWPSTCYNQ**: 84 SER.**DEQ**: 85 SER, 135 TYR, 137 GLN, 167 ARG, 197 GLN.
**Lopinavir**	58GLU, 60ALA, 61TYR, 78ILE, 80*ASP, 139TYR, 140CYS, 141GLU, 176GLU, 178SER, 179LEU, 183GLN, 315GLY, 317THR, 318PHE, 320HIS, 341MET, 342ASN, 374SER, 376ILE, 424SER, 426TRP, 427CYS, 428LYS, 439ILE	58GLU, 60ALA, 61TYR, 78ILE, 80*ASP, 139TYR, 140CYS, 141GLU, 176GLU, 178SER, 179LEU, 183GLN, 323GLY,324SER, 325THR, 326PHE, 328HIS, 349MET, 350ASN, 382SER, 384ILE, 430LYS, 432SER, 434TRP, 436LYS, 447ILE.

Variations in the interacting amino acids speculates that a modulation in the activity of plasmepsin-V might have resulted from imperfect duplicate mutations.

**Table 2:** The comparative docking score of true PEXEL with *Pv*PM-V Sal-1 (wild) and *Pv*PM V- Ind (mutant).

Gene	PEXEL sequences*	Position	Global energy (Wild) (*Pv*PM-V Sal-1)	Global energy (Mutant) (*Pv*PM-V IND)	ACE Native (*Pv*PM-V Sal-1)	ACE Mutant (*Pv*PM V IND)
PVX_102130	ssslcrnisniaekh**KSLMQ**eckekdnnlpnaltn	171–175	−56.26	−65.32	−17.64	−4.72
PVX_107755	eyevklikdadddny**KNICD**iecgsdictndvkai	79–83	−50.44	−17.90	−9.99	−13–32
PVX_108770	eaddetisestiqgq**RIIFE**hlpayifeqklkeda	27–31	−60.33	−77.43	−11.14	−12.08
PVX_09447	vestatveeikksyk**KIILQ**yhpdknshlseeeqk	30–34	−66.04	−63.55	−8.33	−12.79
PVX_115455	sdadafqseltlpng**RTLAE**ketskaqeegfftfe	85–89	−65.64	−36.21	−12.14	−7.90

* The five true PEXEL used for the docking studies displaying the gene number and position of PEXEL motif on the gene. The true PEXEL used in this study tightly follows the true PEXEL formula i.e. [KR][GAVLIMFWPSTCYNQ][LI][GAVLIMFWPSTCYNQ] [DEQ]--.

**Table 3:** The comparative docking score of loose PEXEL with *Pv*PM-V Sal-1 (wild) and *Pv*PM-V- Ind (mutant).

Gene	PEXEL sequences*	Position	Global energy (Wild) *Pv*PM-V Sal-1	Global energy (Mutant) *Pv*PM-V IND	ACE Native *Pv*PM-V Sal-1	ACE Mutant *Pv*PM-V IND
PVX_118695	llkdkriqkkinkml**KGIKQ**hgqmndlrfngnidk	113–117	−63.24	−89.15	−6.97	−4.88
PVX_112630	lyylhthilkdltly**KKLD**Envkdpskiktecsfl	37.41	−69.85	−78.82	−12.11	−15.21
PVX_107750	nnkcncahnivelym**KYIDD**crrgvntefcneldn	173–177	−57.54	−84.02	−11.33	−11.08
PVX_104190	skkddeikhlcskfl**RNLKD**lknekatyddkhryl	57–61	−75.92	−60.38	−5.41	−8.06
PVX_103660	pdkelllnigylqei**KDLFD**ffedynqmkkeiian	166–170	−91.1	−79.13	−17.74	−7.86

* The loose PEXEL used in this study follows the PEXEL formula i.e. {[KR].[LI].[DEQ]}, where capital letters denote individual amino acids, multiple amino acids in brackets[XZ] represent ambiguity in pattern and full stop (.) means any amino acid.

Various reports from India have shown lower sensitivity of rapid diagnostic tests (80–85%) [42] which are based on erythrocytic stage antigens. This could be a result of this type widespread plasmepsin-V polymorphism or selection of this polymorphism by impairing diagnosis. Plasmepsin-V may be a unique drug target, as it is conserved in all *Plasmodium* species and is also a single copy essential gene. The consensus architecture of PEXEL side chains can be used to design a novel inhibitor/pharmacophore specific to *Pv*PM-V. Similar polymorphisms may be screened in *P. falciparum*/cultivable parasites as it has not been done so far & could reveal mutational impact of *Pv*PM-V on antigenic profile of mutants.

### Conclusions

Genetic polymorphism of the *Pv*PM-V could be a novel tactic to change antigenic profile as plasmepsin-V has been shown to be key enzyme for antigenic protein export. The omnipresence of this imperfect duplicate insertions type mutations in different geographical regions of India (*Pv*PM-V-Ind) could imply that either *Plasmodium vivax* infections are all evolved from single ancestor isolated from rest of the world or *Plasmodium vivax* with this polymorphism is more virulent and dominating emerging infections. Sequence analysis suggests importance of this gene in *Plasmodium* genera as it is well conserved in the same but no conservancy with other closely related organisms. Overriding host cellular functions by exporting as many as 200–300 proteins are a characteristic feature of *Plasmodium* sp. [43], [45] thus a conservancy and essentiality of this gene is due to this specialized function. Exported proteins, as identified by the PEXEL motif, play a major role in *Plasmodium* virulence and facilitate the parasite's survival in the host cell. Polymorphism in *Pv*PM-V-Ind gene could possibly have a wider impact by favoring or limiting export of certain PEXEL proteins thereby changing the antigenic profile. Comprehensive knowledge of their diversity and evolution will help to unravel the emergence of the high pathogenicity of *P. vivax*, and may allow the identification of novel targets for malaria therapy.
